# The general movement assessment in non-European low- and middle-income countries

**DOI:** 10.11606/S1518-8787.2018052000332

**Published:** 2018-01-29

**Authors:** Iris Tomantschger, Dafne Herrero, Christa Einspieler, Cristina Hamamura, Mariana Calil Voos, Peter B Marschik

**Affiliations:** I Medical University of Graz. Research Unit Interdisciplinary Developmental Neuroscience. Department of Phoniatrics. Graz, Austria; II Universidade de São Paulo. Faculdade de Saúde Pública. Departamento de Saúde Materno Infantil. São Paulo, SP, Brasil; III Universidade de São Paulo. Faculdade de Medicina. Departamento de Fisioterapia, Fonoaudiologia e Terapia Ocupacional. São Paulo, SP, Brasil; IV Karolinska Institutet. Center of Neurodevelopmental Disorders. Stockholm, Sweden

**Keywords:** Infant, Premature, Cerebral Palsy, Psychomotor Disorders, Psychomotor Performance, Motor Activity, Disability Evaluation

## Abstract

Abnormal general movements are among the most reliable markers for cerebral palsy. General movements are part of the spontaneous motor repertoire and are present from early fetal life until the end of the first half year after term. In addition to its high sensitivity (98%) and specificity (91%), the assessment of general movements is non-invasive and time- and cost-efficient. It is therefore ideal for assessing the integrity of the young nervous system, most notably in lowresource settings. Studies on the general movements assessment in low- and middle-income countries such as China, India, Iran, or South Africa are still rare but increasing. In Brazil, too, researchers have demonstrated that the evaluation of general movements adds to the functional assessment of the young nervous system. Applying general movements assessment in vulnerable populations in Brazil is therefore highly recommended.

## INTRODUCTION

One of the most challenging tasks for medical practitioners is to identify specific risk factors in early infancy and to reliably predict impairment that manifests later in life. Cerebral palsy (CP) is one such condition that usually manifests before 18 months of age^42^, with an overall prevalence of 2.11 per 1,000 live births^37^, increasing to over 10% with decreasing gestational age^29^. As CP affects a number of functional and medical domains, it poses various challenges to affected children, their families, and healthcare, especially in low- and middle-income countries (LMIC), according to the World Bank country classification^48^.

There is increasing evidence for the importance of early intervention in children with motor delays. Simply put: the sooner the intervention, the better the outcome^35^. But CP diagnosis can be tedious and difficult, particularly when human and financial healthcare resources are limited. The LMIC are struggling to provide expensive medical equipment for diagnoses, such as magnetic resonance imaging, computed tomography or cranial ultrasonography. The general movement assessment (GMA, see below) is cost- and time-efficient. Due to its high predictive value (sensitivity 98% [95%CI 74–100]; specificity 91% [95%CI 83–93])^4^, it is used by an ever-increasing number of health professionals around the world for early identification of infants with a high risk for CP^4^ ^,^ ^14^.

### What are General Movements?

Fetuses and young infants display a large repertoire of spontaneous movements such as stretching, yawning, twitching, and a specific motor pattern commonly known as general movements (GM). GM are complex and involve the entire body, notably arm, leg, neck, and trunk movements. They include rotations and vary in speed, intensity, and direction, which makes them appear elegant and fluent. GM emerge at nine weeks postmenstrual age (fetal GM) and continue after birth, with unchanging characteristics at first^13^. So-called preterm GM show no difference to fetal GM. GM that emerge around the term and during the first two months postterm are called writhing movements (WM) – ellipsoidal movements characterized by a small to moderate amplitude and low to moderate speed. At a postterm age of three to five months, WM are gradually replaced by small movements of the neck, trunk, and limbs, which are commonly referred to as fidgety movements (FM)^18^. Finally, with the onset of intentional and anti-gravity movements, GM gradually disappear towards the end of the first half-year of life^14^ ^,^ ^15^.

### The General Movement Assessment

Evaluating the quality of age-specific GM by GMA^15^ mainly serves to predict or rule out neurological impairments that become manifest only later in life (e.g., CP). In a systematic review of assessment techniques for predicting CP, GMA was found to be better suited than cranial ultrasound, neurological examination, or even magnetic resonance imaging^4^. With summary estimates for sensitivity and specificity of 98% (95%CI 74–100) and 91% (95%CI 83–93), respectively, Bosanquet et al.^4^ clearly found GMA to be the method of choice.

Applying GMA is straightforward: for a reliable assessment (kappa values ranging from 0.89 to 0.93^15^), a 3- to 5-minute video of the infant is recorded following few basic principles (e.g., infant lying in supine position, comfortably dressed, not crying)^12^. Performed by trained GM scorers, the assessment as such can be carried out on site^26^ or elsewhere; it is time-efficient and incurs minimal costs^12^. Scorers classify GM as “normal” or “abnormal”. Abnormal preterm GM and WM can be either classified as the following: a) poor repertoire (low complexity and variability, monotony); b) cramped-synchronized (no smoothness, simultaneous contraction and relaxation of the limbs); or c) chaotic (no smoothness, chaotic and abrupt movements of large amplitude)^14^ ^,^ ^15^.

Abnormal FM can be classified as absent or abnormal in terms of exaggerated jerkiness and speed. Already in 1997, Heinz Prechtl et al.^41^ demonstrated that normal FM are a highly reliable marker for a normal neurological development even if the medical history and cranial ultrasound had indicated a high risk for maldevelopment. An absence of FM, by contrast, indicates a neurologically adverse development even in infants with no structural impairment^41^. These findings have been repeatedly confirmed all over the world^2^ ^,^ ^9^ ^,^ ^49^ ^,^ ^50^, with a sensitivity of 91%–98% and a specificity of 81%–91%^4^ ^,^ ^8^.

Although GMA is based on visual Gestalt perception, attempts were recently made to use computer-based analysis^1^ ^,^ ^17^ ^,^ ^34^.

### Early Specific Markers for Cerebral Palsy: The Benefits of General Movement Assessment

Already at late preterm age and around term (i.e., at a term-equivalent age) crampedsynchronized GM, if present for several weeks, were found to be highly predictive for spastic CP^15^ ^,^ ^16^ ^,^ ^21^. At the age of three to five months, it is possible to predict both bilateral and unilateral CP by GMA. Most individuals show no FM, but those who are to develop unilateral CP show initial asymmetries in isolated wrist and finger movements^11^ ^,^ ^25^ ^,^ ^27^. Poor repertoire of GM at term age followed by an absence of FM and circular arm movements can be seen as early markers for dyskinetic CP^13^ ^,^ ^16^. A detailed assessment that goes beyond standard GMA can indicate the severity of CP^16^. Applying Prechtl's optimality concept^40^, Einspieler et al. presented a motor optimality score based both on standard GMA^14^ and on the assessment of other postures and movements than FM. A low motor optimality score was associated with a limited functional mobility and activity^7^ ^,^ ^49^.

### General Movement Assessment in Asian and African Low- and Middle-Income Countries

Its straightforward applicability makes GMA an ideal tool for assessing the young nervous system, all the more so in low-resource settings. But how widespread is GMA in non-European LMIC? To get an idea of the current literature on GMA published in non-European LMIC, we conducted a literature research in PubMed and – for broad coverage – Google Scholar, based on the key terms “general movements” and “general movements assessment”. We then selected English publications with a principal investigator based in an LMIC, according to authorship or the study conducted by them (Box).

**Box:** General movements in non-European low- and middle-income countries.

Reference	Country (World Health Organization region^47^, World Bank income group^48^)	Cohort	General movements assessment	Outcome (age at follow-up; measures)	Main results
Yang et al.^49^ (2012)	China (South-East Asia, upper middle income)	79 children with cerebral palsy (32 of them born preterm)	FM, MOS	2–5 years; gross motor function classification system ^38^	Only 1 infant had developed FM. Children with a low MOS have a limited functional mobility and activity at 2 to 5 years.
Ma et al.^32^ (2015)	China (South-East Asia, upper middle income)	285 preterm infants with (n = 145) resp. without (n = 140) early intervention	writhing movements, FM	14 weeks postterm age; general movements assessment	Cramped-synchronized GM (though not poor-repertoire GM) were associated with lower birth weight and lower gestational age. The intervention resulted in an improvement of GM at 3 to 5 months, especially in preterm infants born at < 32 or > 34 weeks.
Zang et al.^51^ (2016)	China (South-East Asia, upper middle income)	74 very low birth weight infants	FM MOS	12 months; PDMS-2^22^	Both absent FM and a lower MOS were associated with a poor gross and fine motor performance.
Adde et al.^2^ (2016)	India (South Asia, lower middle income)	243 very low birth weight infants	FM MOS	12 months; PDMS-2^22^	Absent or abnormal FM and an abnormal concurrent motor repertoire were associated with a lower gross motor and total motor quotient.
Soleimani et al.^45^ (2015)	Iran (Eastern Mediterranean, upper middle income)	15 infants born at or near term with perinatal asphyxia	FM	12–18 months; Infant Neurological International Battery^20^	The presence or absence of FM was associated with the outcome (sensitivity: 0.80; specificity 1.00).
Burger et al.^9^ (2011)	South Africa (Africa, upper middle income)	115 infants with a birth weight ≤ 1.250g	FM	12 months; PDMS-2^22^, AIMS^39^, neurological assessment^3^	There was a significant association between FM and the outcome at 12 months.
Garcia et al.^24^ (2004)	Brazil (Americas, upper middle income)	40 preterm infants with a gestational age < 35 weeks	preterm GM, writhing movements, FM	Follow-up every 3 months until 24 months; neurological examination and DDST^23^	Abnormal GM were associated with brain injuries and neurological outcome. Normal GM were associated with normal neurological outcome.
Manacero et al.^33^ (2012)	Brazil (Americas, upper middle income)	37 preterm infants born at < 34 weeks	preterm GM	14 months; test of infant motor performance^10^; AIMS^39^; pediatric evaluation of disability inventory^28^	There was no relationship between GM and test of infant motor performance; pre-term infants with cramped-synchronized GM had a lower AIMS centile rank than those with poor-repertoire or normal GM.

AIMS: Alberta Infant Motor Scale; DDST: Denver Developmental Screening Test; FM: fidgety movements; GM: general movements; MOS: motor optimality score; PDMS-2: Peabody Developmental Motor Scales

In Asia, three Chinese studies^32^ ^,^ ^49^ ^,^ ^51^, one study conducted in India^2^, and one Iranian study^45^ met the inclusion criteria. Yang et al.^49^ conducted a longitudinal assessment in a large number of children, confirming that the absence of FM was a significant marker of CP. It was also one of the first studies to find that a detailed assessment of postures and movements other than FM helps to predict CP severity. Although more than 100,000 infants with very low birth weight are born annually in China^31^, studies on their early motor performance are still scarce. Recently, Zang et al.^51^ demonstrated that both absent FM and a low MOS were associated with a poor or even very poor gross and fine motor performance at 12 months corrected age. Such an early identification enables early intervention programmes before pathological features become manifest. In Jinan and Binzou, Ma et al.^32^ showed that early intervention by auditory stimulation, visual stimulation, tactile stimulation, vestibular motion stimulation, pediatric gymnastics, or hydrotherapy improves GM. Apart from these publications, a considerable number of Chinese rehabilitation doctors, neurologists, pediatricians, and physiotherapists constantly exchange their experiences with GMA online (http://www.gmshome.cn). Another study on VLBW infants and their GM was recently conducted in India, applying both visual perception-based and computer-based GMA^2^. Similar to Zang et al.^51^, the Indian study showed that absent or abnormal FM and a monotonously abnormal movement character was associated with lower motor quotients at 12 months corrected age. The computer-based GMA confirmed that the variability of the spatial center of motion was higher – thus indicating absent FM – in children with lower motor quotients^2^.

One of the recent studies on GMA was carried out in Iran. It confirmed the importance of FM observation: according to Soleimani et al.^45^, absent FM indicated a high risk for maldevelopment in a group of infants with perinatal asphyxia.

A similar association between FM and the 12-month outcome was reported from South Africa. In a group of 115 very low birth weight infants, absent FM identified individuals who would perform poorly at the neurodevelopmental and motor assessments around nine months later^9^.

### General Movement Assessment in Brazil

According to the Brazilian Ministry of Health^6^, pre- and perinatal care in the country is still insufficient, with significant impacts on mother-child health. Poor environmental sanitation and malnutrition are the main causes of Brazil's infant mortality (infant mortality rate in 2015: 13.82 deaths per 1,000 live births)^30^ and early childhood diseases^46^. Preventive healthcare and early diagnosis of neurodevelopmental disorders are not yet common in the whole country, especially among the vulnerable population. Childhood disabilities are mainly attributable to a poor economic state in association with the parents’ low educational status and nutritional stunting^44^ ^,^ ^46^. In order to improve the situation, social protection initiatives for infants and young children with an increased risk for developmental delay or impairment have been implemented. Government grants facilitate the access to healthcare, education, transportation, and recreation^6^. A major aim is to improve child development, but when the diagnosis is delayed, many families have no access to most healthcare services and health promotion strategies.

An effective strategy to identify developmental delay is to conduct consecutive assessments^36^. GMA has so far been performed by a group of specifically trained Brazilian therapists. They apply GMA in maternity wards and hospitals (mainly secondary and tertiary care), but few scientific studies on GM are published^5^ ^,^ ^24^ ^,^ ^33^ ^,^ ^43^. In 2008, Santos et al.^43^ discussed various assessment techniques for preterm infants and found GMA to be a reliable and efficient evaluation method (sensitivity 100%, specificity 96%, inter-scorer reliability 92% to 97%). Four years earlier, Garcia et al.^24^ had demonstrated, in a sample of 124 preterm infants born in São Paulo, that normal GM were highly predictive of a normal outcome, while only a few infants with abnormal GM developed normally. Furthermore, the authors reported on a high sensitivity (86%) but lower specificity (53%) for GMA, whereas the cranial ultrasound examination revealed the opposite (sensitivity 60%; specificity 87%). They concluded that both assessment tools should be combined^24^. In 2012, Manacero et al.^33^ questioned whether a single preterm assessment of GM could predict the neurodevelopmental outcome at an age of 14 months. The study was carried out in Porto Alegre, RS, Southern Brazil, and comprised of 37 preterm infants whose GM were assessed at 34 weeks postmenstrual age. Children with cramped-synchronized GM had lower percentile ranks in the Alberta Infant Motor Scales at 14 months than children with poor repertoire or normal GM. The study showed that a single assessment of GM during preterm age was only fairly to moderately associated with the motor outcome at 14 months^33^. In addition, we know that at school age the cognitive and behavioral disabilities can be evidence in infants that were born premature. Only recently, the GMA has also shown its merit for identifying preterm infants at risk for cognitive dysfunction. A normalization of GM before or at term age was associated with higher intelligence quotients at school age compared to a normalization of GM around three to four months postterm age^19^. This clearly indicates the need for serial GMA. Recently, the very first infants intrauterinely exposed to Zika Virus have been evaluated by GMA. The analysis revealed an exceedingly high percentage of abnormal GM^5^.

### Future Prospects for General Movement Assessment in Brazil

The School of Public Health at the Universidade de São Paulo has recently proposed various studies based on GMA. Identifying high-risk infants at an early stage facilitates the implementation of public health follow-up strategies to provide early care. The proposed studies will comprise infants who: are from families with a vulnerable social background; were intrauterinely exposed to substance abuse; were intrauterinely exposed to malaria or infants with neonatal malaria (both are still common in the indigenous population in northwestern Brazil); or have Down syndrome. In addition, an ongoing collaboration between the University of California, Los Angeles (USA), the Fundação Oswaldo Cruz in Rio de Janeiro, the Universidade de São Paulo, and the Medical University of Graz (Austria) revealed first insights, using GMA, into the early neurological development of infants intrauterinely exposed to Zika Virus^5^. These ongoing projects, together with the annually organized GMA training courses, will expand the use of GMA in high-risk populations in Brazil. Clinically, GMA can assist in the prediction of cerebral palsy, which allows for the benefits of early intervention to be provided.

The ongoing studies in Brazil will be joint projects of experts of various scientific disciplines in order to strengthen research on child development in vulnerable populations. In addition to the Brazilian researchers’ efforts, a research unit in Graz, Austria, is currently working on the smartphone-based mobile solution “GMApp” (http://gmapp.idn-research.org), which will significantly facilitate GMA in remote areas. Hopefully, the new technology will gain a substantial foothold in Brazil.

## CONCLUSION

Developmental assessment based on GM considerably facilitates infant screenings aimed at early detection of developmental abnormalities. However, the use of GMA in LMIC such as Brazil still needs a lot of promotion. The objective remains to initiate early intervention and improve future prospects for children at risk for CP worldwide.
